# The safety and outcomes of acutely ruptured intracranial aneurysms with incomplete occlusion after coiling: a case-control study

**DOI:** 10.1186/s12883-020-01991-y

**Published:** 2020-11-11

**Authors:** Jianhe Yue, Yuan Xie, Xiaolin Zhang, Yongxiang Jiang, Weifu Chen, Ying Ma, Yuan Cheng

**Affiliations:** Department of Neurosurgery, Chongqing Medical University Affiliated Second Hospital, No.76 Linjiang road, Yuzhong District, Chongqing, 400010 China

**Keywords:** Ruptured intracranial aneurysms, Incomplete occlusion aneurysms, Stent-assisted coiling, Complications, Outcomes

## Abstract

**Background:**

Incomplete aneurysmal occlusion is a common feature of immediate posttreatment angiography. The safety and outcomes of acutely ruptured intracranial aneurysms (RIAs) with incomplete occlusion after stent-assisted coiling (SAC) and no-stent coiling (NSC) have not been well clarified. Progressive occlusion of stents can promote the complete occlusion of intracranial aneurysms (IAs), but it remains to be determined if progressive occlusion in acutely RIAs with incomplete occlusion after coiling may be enhanced by protective stenting. This study aimed to evaluate the safety and outcomes of those aneurysms after SAC and NSC; And to discover whether the stents can promote progressive aneurysm occlusion in such lesions or not.

**Methods:**

We reviewed 199 patients with acutely RIAs underwent endovascular coiling and developed incomplete occlusion in the past seven years. The patients’ clinical and imaging information were recorded and analyzed. Univariate and multivariate analyses were performed to determine the association of recurrence rate with potential risk factors.

**Results:**

SAC group had wider aneurysms neck (3.471 mm vs 2.830 mm, *P* = 0.009) and smaller dome-to-neck ratio (1.536 vs 2.111, *P* = 0.001) than in NSC group. There was no significant difference between the two groups in total procedure-related complications rate (31.7% vs 23.5%, *P =* 0.195), procedure-related mortality (6.9% vs 2.0%, *P =* 0.170) and modified Rankin Scale (mRS) score at 6-month follow-up (*P* > 0.05). However, SAC group had significantly higher ischemic complications rate (21.8% vs 8.2%, *P* = 0.007) and complete occlusion rate (65.6% vs 48.3%, *P* = 0.020), and lower recurrence rate (15.6% vs 28.1%, *P* = 0.042) than NSC group based on 6-month follow-up angiograms. Additionally, Multivariable analysis showed NSC was an independent risk factor for aneurysm recurrence (Odds Ratio [OR]: 4.061; *P* = 0.018).

**Conclusions:**

Acutely RIAs with incomplete occlusion after SAC is associated with higher complications rate and mortality, but has an acceptable safety profile and similar clinical outcome compared to NSC, as well as gives patients superior angiography outcome by progressive occlusion of stents.

## Background

Despite advances achieved in endovascular techniques, incomplete occlusion is a common feature of immediate posttreatment angiographic studies [1]. Yang et al. [2] reported that initially incomplete occlusion (Modified Raymond-Roy Classification II or III, MRRC II or III) of SAC and NSC were achieved 48.3% vs. 35.7% respectively of the acutely RIAs. The incomplete aneurysm occlusion was usually regarded as a risk or an independent risk factor of aneurysm recurrence or rebleeding [3, 4].

Fortunately, several studies had revealed the capability of stent assistance to augment the progression from incomplete occlusion (MRRC II or III) to complete occlusion (MRRC I) [1, 5–7]. Other study held the doubtful position that progressive aneurysm occlusion was compensated by the progressive regrowth of aneurysms [8]. And, most of previous studies mainly investigated the unruptured intracranial aneurysms (UIAs).

For acutely RIAs, it remains to be determined if progressive occlusion in such lesions after coiling may be enhanced by protective stenting. The safety and outcomes of acutely RIAs with incomplete occlusion after SAC and NSC have also not been well clarified. Thus, we performed this retrospective study to compare the safety and outcomes of those aneurysms after SAC and NSC, and to elucidate progressive aneurysm occlusion of stents in such lesions.

## Methods

According to the agreement approved by the Institutional Review Board. Two independent investigators (Y. JH, X. Y.) retrospectively reviewed the angiography and clinical data of 199 patients treated with endovascular coiling with/without stent for acutely RIAs with incomplete occlusion during 7 years between January 2012 and May 2019.

### Patient selection and population

Inclusion criteria for this study were:

1. Ruptured saccular aneurysms treated with SAC or NSC;
2. Acutely RIA, which was defined as an aneurysm treated no more than 28 days after the initial rupture;
3. Incomplete occlusion aneurysms were defined as MRRC II or III based on immediately postoperative angiograms.

Exclusion criteria for this study were:

1. Aneurysms other than saccular—that is dissecting, fusiform, traumatic, and blood blister-like;
2. Aneurysms treated with “special” treatment strategies—that is, staged stent placement and stent placement alone;
3. Aneurysms treated with clipping or assisted coiling with balloon or flow diverters;
4. Patients with multiple aneurysms, the ruptured one cannot be certainly identified;
5. Patients with multiple aneurysms and treated both the ruptured and unruptured one.

### Procedure technique

Treatment decisions of clipping or coiling concerning patient comorbidities, clinical status, surgical feasibility, and grade of hemorrhage were made in consensus among the neurosurgeons and neuro-interventionalists. Stent placement was determined according to the aneurysm feature and the intraoperative requirement when the endovascular coiling was determined as the treatment plan for patients. All operations were performed under general anesthesia. A 6-Fr or 8-Fr guiding catheter was positioned in the internal carotid artery via the common femoral artery for anterior circulation aneurysms or in the vertebral artery for posterior circulation aneurysms. Types of stents used in this procedure included the Leo stent (Balt, Montmorency, France), the Enterprise stent (Codman Neurovascular, USA), and the LVIS stent (Microvention, California, USA). Stents were chosen according to the surgeon’s preference concerning anatomical variations.

### Anticoagulation and antiplatelet management

A femoral artery catheter was inserted in a standard fashion, and the femoral artery catheter performed the systemic heparinization for all patients. Glycoprotein IIb/IIIa inhibitor (Tirofiban; Grand Pharma) was used to prevent thrombosis when the stents were opened. The loading dose of the first intravenous infusion was 0.3 mg (intravenous infusion time was 3 min), and then injected intravenously at 0.3 mg per hour by a trace syringe pump. If acute thrombosis occurred during the procedure, additional doses of tirofiban would be used. If there was no thrombosis or hemorrhagic events happened during the process, tirofiban will be discontinued after the operation finished. The postoperative antiplatelet regimen has been aspirin 100 mg daily for 6 months and clopidogrel 75 mg daily for 4 weeks. Moreover, the first 3 days after surgery, the low-molecular-weight heparin (subcutaneous injection 2000iu; q12h) is used in combination with antiplatelet agents.

### Clinical and angiographic follow-up

The safety was measured by total procedure-related complications, mortality, mRS score at discharge, and the 6-month follow-up. Based on the mRS score at the 6-month follow-up, a favorable clinical outcome was defined as mRS score 0–2 and an unfavorable outcome as mRS score 3–5. Immediately postoperative angiograms were used to analyze in terms of the degree of IAs obliteration by using the MRRC (I, complete obliteration; II, residual neck; IIIa, residual aneurysm with contrast within coil interstices; IIIb, residual aneurysm with contrast along aneurysm wall). The angiography follow-up results obtained with digital subtraction angiography (DSA) or three-dimensional contrast-enhanced magnetic resonance angiography (3D CE-MRA). In patients undergoing 3D CE-MRA at 6 months, the findings with not-stable aneurysms (any change) would be confirmed by DSA. And these results were classified into 4 categories when compared with the immediate embolization degree:

1. occluded, defined as no contrast material filling into the aneurysm sac and neck;
2. improved, defined as decreased contrast material filling into the aneurysm sac or neck;
3. stable, defined as unchanged contrast material filling into the aneurysm sac or neck;
4. recurrent, defined as increased contrast material filling into the aneurysm sac.

The same two physicians who evaluated the immediate postoperative angiograms also evaluated the angiography follow-up outcome.

### Statistical analysis

Statistical analysis was performed by using SPSS version 22.0 software (SPSS. Chicago) by two authors. Data are presented as the mean and range for continuous variables, and frequency and percentage for categorical variables. Mann-Whitney U tests were used for non–normally distributed but continuous variables. Pearsonχ^2^ or Fisher exact test was used for categorical variables. Univariate analysis and multivariate analysis were performed to determine the association of recurrence rate with potential risk factors. The univariate analysis cutoff for inclusion in the multivariate analysis was *P* < 0.05. The level of statistical significance was set at P < 0.05.

## Results

### Baseline characteristics

Of the 199 patients, 101 patients were treated with SAC, and 98 patients were treated with NSC. The patient’s and aneurysm’s baseline characteristics were summarized in Tables 1 and 2. There was no significant statistical differences in patient age, sex, medical history, personal history, Glasgow coma scale (GCS) score, Hunt-Hess grade, Fisher grade, and aneurysm size in the 2 groups (*P* > 0.05). However, there were a significantly wider aneurysm neck and smaller dome-to-neck ratio in the SAC group (*P* < 0.05). Additionally, the SAC group had a significantly higher incidence in internal carotid aneurysms (ICA) than the NSC group (*P* = 0.034).

**Table 1:** Patient Baseline Characteristics *N*(%)

	SAC(*n* = 101)	NSC(*n* = 98)	*P* value
Age (years),mean ± SD	54.94 ± 11.33	54.50 ± 10.58	0.612^c^
Female	71 (70.3)	67 (68.4)	0.798^a^
Hypertension	41 (40.6)	31 (31.6)	0.188^a^
Hypertension ≥5 years	22 (21.8)	15 (15.3)	0.658^a^
Diabetes Mellitus	0 (0)	4 (4.1)	0.057^b^
Coronary heart disease	1 (1)	3 (3.1)	0.364^b^
Stroke history	0 (0)	2 (2.0)	0.149^a^
Smoking history	22 (21.8)	23 (23.6)	0.776^a^
Smoking class (years^a^cigarettes/day ≥400)	15 (14.9)	19 (19.4)	0.260^a^
Drinking history	8 (7.9)	7 (7.1)	0.835^a^
GCS score	13.71 ± 2.16	13.33 ± 2.70	0.427^c^
Hunt-Hess grade			
I	16 (15.8)	22 (22.4)	0.597^c^
II	68 (67.3)	58 (59.2)	
III	13 (12.9)	11 (11.2)	
IV	4 (4.0)	7 (7.1)	
Fisher grade			
I	19 (18.8)	21 (21.4)	0.318^c^
II	43 (42.6)	48 (49.0)	
III	23 (22.8)	10 (10.2)	
IV	17 (16.8)	19 (19.4)	

Data are presented as number (%) or mean ± SD unless otherwise indicated

*SAC* stent-assisted coil embolization, *NSC* no-stent coil embolization, *GCS* Glasgow Coma Scale

^a^ Calculated using chi-square test

^b^ Calculated using Fisher’s exact test

^c^ Calculated usingnon-parametric test

**Table 2:** Aneurysm Baseline Characteristics *N*(%)

	SAC(n = 101)	NSC(n = 98)	*P* value
Neck size, mean ± SD, mm	3.471 ± 1.845	2.830 ± 1.147	0.009^c^§
Maximize size, mean ± SD, mm	5.250 ± 2.937	5.770 ± 2.563	0.079^c^
Dome-to-neck ratio, mean ± SD	1.536 ± 0.462	2.111 ± 0.578	0.001^c^§
Aneurysms size ≥10, mm	3 (3.0)	9 (9.2)	0.209^ab^
5 ≤ Aneurysms size < 10, mm	45 (36.2)	48 (48.5)	0.820^a^
Aneurysms size < 5, mm	46 (45.5)	50 (50.5)	0.769^a^
Location			
ICA	14 (13.9)	5 (5.1)	0.034^a^§
PComA	39 (38.6)	43 (43.9)	0.451^a^
ACA	4 (4.0)	4 (4.1)	1.000^b^
AComA	34 (33.7)	30 (30.6)	0.645^a^
MCA	6 (5.9)	8 (8.2)	0.540^a^
PC	4 (4.0)	7 (7.1)	0.326^a^
Parent artery configuration			
Bifurcation	84 (83.2)	89 (90.8)	0.110^a^
Side wall	17 (16.8)	9 (9.2)	
Second rupture	11 (10.9)	12 (12.2)	0.765^a^
Intracranial hematoma	6 (5.9)	13 (13.3)	0.079^a^
Interval between rupture & treatment			
≤ 72 h	42 (41.6)	26 (26.5)	0.025^a^§
3–28 days	59 (58.4)	72 (73.5)	

Data are presented as number (%) or mean ± SD unless otherwise indicated

*SAC* stent-assisted coil embolization, *NSC* no-stent coil embolization, *ACA* anterior cerebral artery, *AComA* anterior communicating artery, *ICA* internal carotid artery, *MCA* middle cerebral artery, *PC* posterior circulation, *PComA* posterior communicating artery

^a^ Calculated using chi-square test

^b^ Calculated using Fisher’s exact test

^c^ Calculated usingnon-parametric test

§ Statistically significant at *p* ≤ 0.05

### Complications

The SAC group had a higher rate than the NSC group in total procedure-related complications (31.7% vs 23.5%, *P* = 0.195) and mortality (6.9% vs 2.0%, *P* = 0.170), but with no statistical difference. Ischemic events (21.8% vs 8.2%, *P* = 0.007) and intraoperative thrombosis (15.8 vs 5.1%, *P* = 0.014) were prominent in the SAC group. Hemorrhagic events (14.8% vs 17.3%, *P* = 0.632) and intraoperative rupture (IOR) rates (11.9% vs 17.3%, *P* = 0.275) between the two groups were comparable. The postoperative thrombosis (5.9% vs 3.1%), postoperative hemorrhage (3.0% vs 0%) and aneurysm rebleeding (5.9% vs 1.0%) in SAC group were slightly higher than in NSC group, but with no statistical difference (*P* > 0.05). The detailed data were shown in Table 3.

**Table 3:** Angiographic And Clinical Outcomes Of SAC And NSC *N*(%)

	SAC(n = 101)	NSC(n = 98)	*P* value
Total procedure-related complication	32 (31.7)	23 (23.5)	0.195^a^
Ischemic	22 (21.8)	8 (8.2)	0.007^a^‡
Intraoperative thrombosis	16 (15.8)	5 (5.1)	0.014^a^‡
Postoperative thrombosis	6 (5.9)	3 (3.1)	0.328^a^
Hemorrhagic	15 (14.8)	17 (17.3)	0.632^a^
Postoperative hemorrhage	3 (3.0)	0 (0)	0.246^b^
Intraoperative rupture	12 (11.9)	17 (17.3)	0.275^a^
Aneurysm rebleeding	6 (5.9)	1 (1.0)	0.119^b^
Procedure-related motality	7 (6.9)	2 (2.0)	0.170^b^
Raymond class			
II	63 (62.4)	61 (62.2)	0.985^a^
III	38 (37.6)	37 (37.8)	
mRS score at charge			
0–2 score	77/94 (81.9)	87/96 (90.6)	0.081^a^
3–5 score	17/94 (19.1)	9/96 (9.4)	
mRS score in 6-month FU			
0–2 score	76/90 (84.4)	80/89 (89.9)	0.277^a^
3–5 score	14/90 (15.6)	9/89 (10.1)	
Complete occlusion in 6-month FU	59/90 (65.6)	43/89 (48.3)	0.020^a^‡
Improved in 6-month FU	8/90 (8.9)	11/89 (12.4)	0.451^a^
Stable in 6-month FU	10/90 (11.1)	10/89 (11.2)	0.979^a^
Recurrence in 6-month FU	14/90 (15.6)	25/89 (28.1)	0.042^a^‡

Data are presented as number (%) or mean ± SD unless otherwise indicated

*SAC* stent-assisted coil embolization, *NSC* no-stent coil embolization, *ACA* anterior cerebral artery, *AComA* anterior communicating artery, *ICA* internal carotid artery, *MCA* middle cerebral artery, *PC* posterior circulation, *PComA* posterior communicating artery

^a^ Calculated using chi-square test

^b^ Calculated using Fisher’s exact test

‡ Statistically significant at *p* ≤ 0.05

We also performed a comparative analysis of complications in early (before 72 h) and late (day 3–28) stage of SAC and NSC group. And there was no difference in different operation timing of the two groups (*P* > 0.05), the data were shown in Tables 4 and 5. Additionally, 11 of 21 patients with intraoperative thrombosis events had successful recanalization by infusion tirofiban. IOR occurred in 29 patients, of whom 7 (24.1%) died, accounting for 77.8% (7/9) of total death events. IOR occurred in 12 patients in the SAC group, of which 5 died (41.7%). IOR occurred in 17 cases in the NSC group, including 2 deaths (11.8%).

**Table 4:** Complications In Early (before 72 h) And Late (day 3–28) Stage Of SAC Group *N*(%)

	Early (*n* = 42)	Late (*n* = 59)	*P* value
Total procedure-related complication	12 (28.6)	20 (33.9)	0.571^a^
Intraoperative thrombosis	7 (16.7)	9 (15.3)	0.848^a^
Postoperative thrombosis	2 (4.8)	4 (6.8)	1.000^b^
Postoperative hemorrhage	2 (4.8)	1 (1.7)	0.569^b^
Intraoperative rupture	5 (11.9)	7 (11.9)	1.000^b^
Aneurysm rebleeding	2 (4.8)	4 (6.8)	1.000^b^
Procedure-related mortality	3 (7.1)	4 (6.8)	1.000^b^

*SAC* stent-assisted coil embolization

^a^ Calculated using chi-square test

^b^ Calculated using Fisher’s exact test

**Table 5:** Complications In Early (before 72 h) And Late (day 3–28) Stage Of NSC Group *N*(%)

	Early (*n* = 26)	Late (*n* = 72)	*P* value
Total procedure-related complication	6 (23.1)	17 (23.6)	0.956^a^
Intraoperative thrombosis	2 (7.7)	3 (4.2)	0.606^b^
Postoperative thrombosis	0 (0)	3 (4.2)	0.563^b^
Postoperative hemorrhage	0	0	–
Intraoperative rupture	4 (15.4)	13 (18.1)	1.000^b^
Aneurysm rebleeding	1 (3.8)	0 (0)	0.265^b^
Procedure-related mortality	1 (3.8)	1 (1.4)	0.462^b^

*NSC* no-stent coil embolization

^a^ Calculated using chi-square test

^b^ Calculated using Fisher’s exact test

### Clinical outcomes

SAC group had higher procedure-related mortality than the NSC group (6.9% vs 2.0%), but without statistical difference (*P* = 0.170). 9 (4.5%) patients died of the two groups. Among these death cases, 7 patients died of IOR (5 in SAC and 2 in NSC), 1 patient died of intraoperative thrombosis which led to massive cerebral infarctions and cerebral hernia, and 1 patient died of postoperative cerebral hemorrhage combined with multiple cerebral infarctions. Clinical evaluation was performed for all 190 surviving patients at discharge based on mRS score, 164 (82.4%) of whom got the favorable clinical outcome (mRS score of 0–2), while the other 26 were dependent (mRS score of 3–5). All 179 patients underwent clinical follow-up after discharge at postoperative 6 months, 156 (87.2%) patients got mRS score 0–2. All 7 patients with aneurysm rebleeding didn’t get admission again and lose further follow-up data (Table 3).

### Angiographic outcomes

According to the MRRC system, a total of 124 patients with MRRC II and 75 patients with MRRC III were analyzed by this study. There was no statistical difference in MRRC based on immediate postoperative angiograms between the two groups. 179 patients got follow-up angiography outcomes at 6-month. Of those, 90 patients (89.1%) treated with SAC and 89 (90.8%) patients treated with NSC got follow-up 6-month. The follow-up angiograms showed that 102 aneurysms (57.0%) were occluded, 19 (10.6%) were improved, 20 (11.2%) were stable, and 39 (21.8%) were recurrent. The complete occlusion rate of SAC and NSC group was 65.6 and 48.3%, respectively, with a significantly statistical difference (*P* = 0.020). And the recurrence rate of SAC and NSC group was 15.6% vs. 28.1%, still with an obvious statistical difference (*P* = 0.042) (Table 3).

Then, the potential risk factors related to the recurrence rates were categorized and analyzed in Table 6. Univariate analysis showed that larger aneurysm size, aneurysms size ≥10 mm, coiling alone were a risk factors for aneurysms recurrence (Table 6). Multivariable analysis showed without stent (OR: 4.061, 95%Confidence Interval [CI]: 1.276–12.926; *P* = 0.018) was an independent risk factor for aneurysm recurrence (Table 7).

**Table 6:** Potential Risk Factors Related To Recurrence Rate *N*(%)

Variable	No Recurrence (*n* = 140)	Recurrence (*n* = 39)	Total	*P* value
Female	97 (69.3)	28 (71.8)	125	0.763^a^
Hypertension	50 (35.7)	14 (35.9)	64	0.983^a^
Hypertension ≥5 years	24 (17.1)	7 (17.9)	31	0.895^a^
Diabetes Mellitus	4 (2.9)	0 (0)	4	0.578^b^
Coronary heart disease	3 (2.1)	0 (0)	3	1.000^b^
Stroke history	2 (1.4)	0 (0)	2	1.000^b^
Smoking history	32 (22.9)	9 (23.1)	41	0.977^a^
Smoking class (years^a^ cigarettes/day ≥400)	25 (17.9)	6 (15.4)	31	0.662^b^
Drinking history	10 (7.1)	3 (7.8)	13	1.000^b^
Aneurysms size ≥10, mm	4 (2.9)	6 (15.4)	10	0.008^b^
5 ≤ Aneurysms size < 10, mm	61 (43.6)	19 (48.7)	80	0.569^a^
Aneurysms size < 5, mm	75 (53.6)	14 (35.9)	89	0.067^a^
ICA	15 (10.7)	2 (5.1)	17	0.370^b^
AComA	49 (35.0)	11 (28.2)	60	0.427^a^
ACA	6 (4.3)	2 (5.1)	8	0.686^b^
PComA	50 (35.7)	15 (38.5)	65	0.887^a^
MCA	12 (8.6)	1 (2.6)	13	0.303^b^
PC	7 (5.0)	2 (5.1)	9	1.000^b^
Parent artery configuration	19 (13.6)	4 (10.3)	23	0.584^a^
Intraoperative rupture (IOR)	17 (12.1)	5 (12.8)	22	1.000^b^
Intraoperative thrombosis	16 (11.4)	4 (10.3)	20	1.000^b^
postoperative thrombosis	6 (4.3)	1 (2.6)	7	1.000^b^
Procedure-related hemorrhagic	2 (1.4)	0 (0)	2	1.000^b^
With stent	76 (54.3)	14 (35.9)	90	0.042^a^‡
Neck size, mean ± SD, mm	3.08 ± 1.64	3.42 ± 1.49	–	0.087^a^
Maximize size, mean ± SD, mm	5.03 ± 2.63	6.41 ± 3.43	–	0.007^a^‡

Data are presented as number (%) or mean ± SD unless otherwise indicated

*ACA* anterior cerebral artery, *AComA* anterior communicating artery, *ICA* internal carotid artery, *MCA* middle cerebral artery, *PC* posterior circulation, *PComA* posterior communicating artery, *IOR* Intraoperative rupture, *FU* follow-up

^a^ Calculated using chi-square test

^b^ Calculated using Fisher’s exact test

‡ Statistically significant at *p* ≤ 0.05

**Table 7:** The Results Of Logistic Regression Analysis

Factors	Odds Ratio	95% Confidence Interval	*P* Value
Aneurysms size ≥10, mm	2.093	0.101–43.259	0.633
Maximize size, mean ± SD, mm	1.044	0.789–1.380	0.765
Without stent	4.061	1.276–12.926	0.018*

Data are presented as number (%) or mean ± SD unless otherwise indicated

* Statistically significant at *p* ≤ 0.05

## Discussion

In this study, we compared the safety, angiography outcome, and clinical outcome of acutely RIAs with incomplete occlusion after SAC and NSC. We first discussed the advantages and indications of using stents for treating acutely RIAs, and then analyzed the reasons for higher complications rate and mortality in the SAC group. Our study had also indicated that the progressive occlusion of stent deployment can promote complete occlusion of acutely RIAs with incomplete occlusion. We were able to detect several risk factors and independent predictors of recurrence.

Clipping and Coiling are 2 accepted treatment strategies for IAs. Clipping has an advantage in treating acutely ruptured wide-necked and complex IAs. It also can improve complete occlusion rate and reduce costs [9–11]. Surgery, however, may also be challenging in some of these lesions, since clips may slip, and surgical access may be limited because of the swelling of the brain in the acute setting of a subarachnoid hemorrhage (SAH). Coiling was associated with a reduced mortality rate and improved outcomes for patients with RIAs according to the results of the International Subarachnoid Aneurysm Trial (ISAT) [9, 12]. And Maud et al. found that coiling has better cost-effectiveness ratio, the costs of coiling would be progressively decreased and eventually reversed as time goes on [11]. Other advantages of coiling are less surgical injury, better cognitive outcome, and lower complication rate [10, 13]. In addition, with the rapid technological advances in endovascular treatment, aneurysms with complex geometry can also be effectively treated with endovascular techniques [2, 3, 14–16]. Therefore, endovascular coiling is becoming a widely accepted treatment modality of RIAs.

There are many endovascular coiling techniques to treat complex and wide-neck IAs. The most common assisted-coiling techniques are balloon, flow diverter (FD) and stent. The balloon-assisted coiling (BAC) provides a contemporary scaffold effect that prevents coil protrusion and is associated with an improved occlusion rate after endovascular therapy in previous studies [17–19]. Wang et al. [16] conducted a meta-analysis comparing BAC and SAC in IAs, and found that SAC achieved a better complete occlusion rate of aneurysms at 6 months or later after the procedure, with comparable complication and retreatment rates. According to previous studies, FD tends to achieve improved occlusion rates at immediate and follow-up angiography [14, 15]. However, the periprocedural complication rate seems to be high. Zhou et al. [20] reported that the complication rate for RIAs was 30.6%% after FD. And FD requires prolonged dual anti-platelet therapy (DAPT), limiting utility in the setting of acute SAH. Additionally, temporary SAC is seemed to be an alternative option for RIAs [21].

Acutely RIAs treated with SAC is still controversial because of concerns about the complexity of the procedure, thrombotic risk, and antiplatelet drugs [22, 23]. These drugs expose patients to an elevated risk of hemorrhagic complications if additional surgical interventions such as decompressive craniectomy, external ventricular drainage (EVD) placement, are required [23, 24]. Moreover, DAPT can increase the risk of all types of bleeding by more than 40% [25]. It is still gradually accepted by neurosurgeons due to its advantages in terms of effectiveness, durability, and facility in treating complex and wide-necked IAs [3, 4, 26]. SAC can also improve aneurysmal complete occlusion rate, reduce recurrence rate, and decrease incidence of rebleeding or retreat rate [27, 28]. Furthermore, SAC brings a comparable complication rate than to NSC [2, 17, 26–29]. And the clinical outcome of the two groups are also comparable, such as a recent systematic review by Zhang et al. [27], shown that favorable clinical outcome rate of SAC and NSC group at discharge and at follow-up were 73.4% vs 74.2, 85.6% vs 87.9% respectively.

In the SAC group of this study, the incidence of ischemic and hemorrhagic complication was 21.8% (22/101), 14.8% (15/101), respectively. It showed a higher incidence than previous systematic reviews. Nishido et al. [22] revealed that the complication rate of stent group was 9.4% (28/299; ischemia: 7.0%; hemorrhage: 2.3%). Bodily et al. [23] reported that total complications rate was 13.6% (39/288) in the stent group (hemorrhage: 8%; ischemia: 5.6%). In several large sample studies, Ryu et al. [30] reported that the event rates of stent group in thromboembolism and intra- and postprocedural hemorrhage were 11.2% (122/1090), 5.4% (59/1090), and 3.6% (39/1090), respectively. In the article by Shapiro et al. [31], the overall procedure complication rate of stent group was 19% (288/1517). In a recent systematic review by Zhang et al. [27], showed that the overall perioperative complications in the SAC group was 20.2% (101/499).

Complications of the stent group in this study was mainly caused by intraoperative thrombosis (15.8%, 16/101) and IOR (11.9%, 12/101). The number one reason of high intraoperative thrombosis events probably was related with the method of antiplatelet administration. Ryu et al. [30] concluded that the antiplatelet regimen used in this study was more likely to bring perioperative thromboembolic complications to patients, Second, the high intraoperative thrombosis events of stent group might be associated with the experience of the endovascular neurosurgeon. The neurosurgeon with little experience may need more endovascular procedure time to place stents, that may increase the incidence of intraoperative thrombosis. These junior neurosurgeons also need to repeatedly retrieve and release stents to ensure that the stents are placed in the optimal position, which may induce vessel endothelium injury, platelet aggregation, vasospasm, or thrombosis. Although, the intraoperative thrombosis rate of the SAC group in this study (15.8%) was consistent with previous literature (5.3–20%) [2, 30, 32, 33], such high incidence of intraoperative thrombosis events still deserved attention and improvement. Third, the poor Plavix response and inadequate use of DAPT may also contribute to the high thromboembolism complications rate of present study. Lee et al. [34] found that 42.9% of their patients had a poor response to clopidogrel, and all thromboembolism events occurred in the poor-response group, which implied that a poor response to antiplatelet medication might induce thromboembolism complications. Piotin et al. [35] and Choi et al. [36] reported that the SAC group had a significantly higher permanent complication rate than the coiling-alone group when DAPT wasn’t used in patients treated with stenting. Therefore, we concluded that the consequences of DAPT’s inadequate use may be similar to that of poor Plavix response or not using of anti-platelet drugs. Additionally, acute SAH is usually regarded as a hypercoagulable state in which the tendency for thrombosis is high [29, 37]. Patients who undergo SAC after period of vasospasm (day 15–20) might have a different complication rate compared to patients treated early with SAC. We therefore performed a comparative analysis of complications in early (before 72 h) and late (day 3–28) stage of SAC and NSC group, and found that timing of treatment has no significantly influence on complications (Tables 4 and 5). These results showed that the SAC is as safe early as well as late after aneurysm rupture.

IOR is a severe complication of coiling and is associated with high rates of morbidity and mortality. According to the review of the Cerebral Aneurysm Re-rupture After Treatment (CARAT) study, the coiling-related IOR occurred in 5% of patients, and IOR was associated with 64% rate of periprocedural death and disability [38]. Recently, Kocur et al. [39] summarized previous literature and showed that the incidence of IOR ranges from 1 to 8.7% and the risk of permanent neurologic disability and death related to IOR is up to 63%. There are many risk factors for the IOR, such as ruptured aneurysms, small aneurysms size, device size, race, and lower initial Hunt and Hess Grade [38–41], even intra-aneurysmal contrast media injection and sudden rise in arterial pressure may also induce IOR [39]. In this study, the IOR occurred in 14.6% of patients, such high IOR rate in the two groups probably contributes to the experience of the endovascular neurosurgeon and the aneurysms status. In the NSC group, the incidence of coil protrusion may higher than SAC group because aneurysm neck lacks stents coverage. Coil protrusion usually requires re-coiling, and a high frequency of repeated coiling may increase the incidence of IOR in the NSC group. It’s worth noting that IOR caused 77.8% (7/9) of all death events in present study. Moreover, the mortality of patients with IOR in the SAC group (41.7%) was higher than that in the NSC group (11.8%). These results revealed that stent deployment may raise the death risk of patients with IOR because tirofiban can affect the physiological hemostasis when aneurysm IOR occurs.

Additionally, some studies even reported that ruptured aneurysm was an independent predictor of complications [35, 37, 42]. Ryu et al. [30] found that the risk of complications rate were higher in the RIAs than in the UIAs group. And Bechan et al. [33] reported that the complication rate of SAC with early adverse events in RIAs was 10 times higher than that in UIAs. And, we found that previous studies included both RIAs and UIAs in reporting the overall complication rate [22, 30, 31]. Therefore, all included aneurysms of this study were RIAs may also be one of the reasons why the complication rate is higher than that in previous literature. The age, sex, underlying disease, past medical history, initial GOS score, Hunt-Hess grade and Fisher grade may also affect the complication rate and clinical outcome of patients with acutely RIAs, but it is comparable between the two groups in this study. There was a significantly wider aneurysm neck and smaller dome-to-neck ratio in SAC group. However, this difference is inevitable, and the original intention of using stents is to treat complex and wide-necked IAs. Many studies reported differences or significant differences in aneurysm neck and dome-to-neck ratio between the two groups [2, 3, 28, 29, 43].

There was no statistical difference in mortality and rebleeding rate between the two groups in this study, and are consistent with previous studies [2, 23, 27–29, 43]. The mRS score 0–2 at discharge and 6-month follow-up of SAC vs NSC was 81.9% vs. 90.6% (*P* = 0.081), 83.3% vs. 89.9% (*P* = 0.244) respectively in this study. Zhang et al. [27] reported similar clinical outcome to our study. We also found that the favorable clinical outcome rates of recent studies were improved than before [12, 19, 27]. The improvement probably attributed to advances in interventional techniques and physicians’ experience in managing SAH patients. In summary, stent placement is associated with a higher rate of complications, mortality and rebleeding rate, but the favorable clinical outcome was still similar to the NSC group.

The use of stent usually brings future superior angiography outcome for patients, even in incomplete occlusion aneurysms [1, 2, 5, 22, 27]. This result has also been confirmed by multivariable analysis of this study. Coil compaction and aneurysm growth are two methods of aneurysm recurrence [44, 45]. Stent placement may increase the capacity of coils to resist the impact of blood flow. Stents can also promote thrombosis within the aneurysmal sac by a scaffold effect, which reduces the recurrence rate [1, 5, 6, 35]. Flow-diversion function and facilitation of endothelialization of stents may also induce changes in intra-aneurysmal hemodynamics and promote delayed occlusion and obtain complete occlusion in the long term [6, 46]. However, our study still has a higher recurrence rate and lower occlusion rate than previous studies both in the SAC and NSC group [1, 5–7]. Furthermore, in several previous studies, progressive complete occlusion of stent placement was achieved 76.5–88.3% in follow-up imaging studies at 6 months [1, 5], which is also higher than 65.6% reported in this study. The first reason is that previous studies mainly described the UIAs, while all included aneurysms in this study were RIAs. RIAs are usually regarded as a predictor of recurrence [2, 35]. Second, the overall statistics of this study show higher recurrence rate and lower occlusion rate compared to literature data probably related to the experience of the endovascular neurosurgeon. Additionally, Bechan et al. [33] reported that the DAPT maybe increase the risk of aneurysm recurrence. Nevertheless, our study found that despite the use of DAPT, the SAC group’s recurrence rate was still lower than in the NSC group, which suggested that the progressive occlusion of stent deployment may not be entirely compensated by the progressive regrowth of aneurysms and the use of DAPT.

Although we conducted some objective and reliable results, and still with some limitations. The major limitation of this study is that this is a retrospective study with no randomization; the patient’s selection of SAC group didn’t establish uniform inclusion and exclusion criteria, so selection bias was inevitable. Additionally, aneurysm characteristics between the SAC group and the NSC group were the slight differences, especially the differences in aneurysm neck and dome-to-neck ratio. Finally, the BAC was not thoroughly considered in deciding the treatment plan because the BAC technique is still developing in our institute and we didn’t have much experience, so the complications of SAC group may overestimate by this study due to patients who are suitable for BAC are selected into the SAC group. Another limitation is that this study had no long-term follow-up results and small sample size.

## Conclusions

The overall procedure-related complications rate and mortality in the SAC group were higher than in the NSC group, but it is still comparable. Moreover, the favorable clinical outcome rate of stents placement was similar to those in no-stent group. These results suggest that acutely RIAs with incomplete occlusion treated by SAC were safe for patients. As well as we performed a comparative analysis of complications in early (before 72 h) and late (day 3–28) stage of SAC and NSC group, and show that the SAC is as safe early as well as late after aneurysm rupture. The follow-up angiography outcome elucidated that stent placement could increase aneurysm occlusion rate, reduce recurrence rate for those patients. And the progressive occlusion of stent deployment may not be entirely compensated by the progressive regrowth of aneurysms and the use of DAPT.

## Data Availability

The datasets used and/or analyzed during the current study are available from the corresponding author on reasonable request.

## Abbreviations

RIAs: Ruptured intracranial aneurysms
SAC: Stent-assisted coiling
NSC: No-stent coiling
IAs: Intracranial aneurysms
mRS: modified Rankin Scale
OR: Odds Ratio
MRRC: Modified Raymond-Roy Classification
UIAs: Unruptured intracranial aneurysms
DSA: Digital subtraction angiography
3D CE-MRA: Three-dimensional contrast-enhanced magnetic resonance angiography
GCS: Glasgow coma scale
ICA: Internal carotid aneurysms
IOR: Intraoperative rupture
CI: Confidence Interval
SAH: Aneurysmal subarachnoid hemorrhage
ISAT: International Subarachnoid Aneurysm Trial
FD: Flow diverter
BAC: Balloon assisted coiling
EVD: External ventricular drainage
DAPT: Dual antiplatelet therapy
CARAT: Cerebral Aneurysm Re-rupture After Treatment
